# Adrenal Angiosarcoma: A Diagnostic Dilemma

**DOI:** 10.7759/cureus.5370

**Published:** 2019-08-12

**Authors:** Nirav Antao, Michael Ogawa, Zarir Ahmed, Jinhua Piao, Nishant Poddar

**Affiliations:** 1 Internal Medicine, St. Louis University School of Medicine, St. Louis, USA; 2 Hematology Oncology, St. Louis University School of Medicine, St. Louis, USA; 3 Pathology, St. Louis University School of Medicine, St. Louis, USA

**Keywords:** adrenal angiosarcoma, immunohistochemistry, aggressive malignancy, adrenal mass, adrenal cancer

## Abstract

Primary angiosarcoma of the adrenal gland is both a rare and aggressive malignancy. Differentiating it from more common adrenal masses such as adrenal adenomas, adrenal cortical carcinomas, and metastatic carcinomas is one of several diagnostic challenges. Immunohistochemical analysis is imperative to arrive at the correct diagnosis. Treatment typically involves surgery and adjuvant chemotherapy, but prognosis remains poor.

## Introduction

Angiosarcoma is a rare high-grade malignancy that originates from the endothelium of blood and lymphatic vessels. It accounts for less than 1% of all soft tissue sarcomas [1]. Skin and soft tissue angiosarcomas are more common than sarcomas that arise in the visceral organs or bone, which are typically sites of metastatic disease [2-3]. Primary angiosarcoma of the adrenal gland is an exceptionally uncommon malignancy with less than 50 cases reported in the literature. Here, we describe the case of a patient who presented with abdominal pain and was discovered to have a large adrenal mass, which upon biopsy, was shown to be epithelioid adrenal angiosarcoma. He underwent surgical resection followed by adjuvant chemotherapy. However, he soon developed recurrent disease with metastatic progression despite multiple lines of chemotherapy, and unfortunately succumbed to this aggressive malignancy. This case is important because it highlights the challenges and potential pitfalls in diagnosing this malignancy, which can often be confused with adrenal cortical carcinoma.

## Case presentation

**Figure 1:**
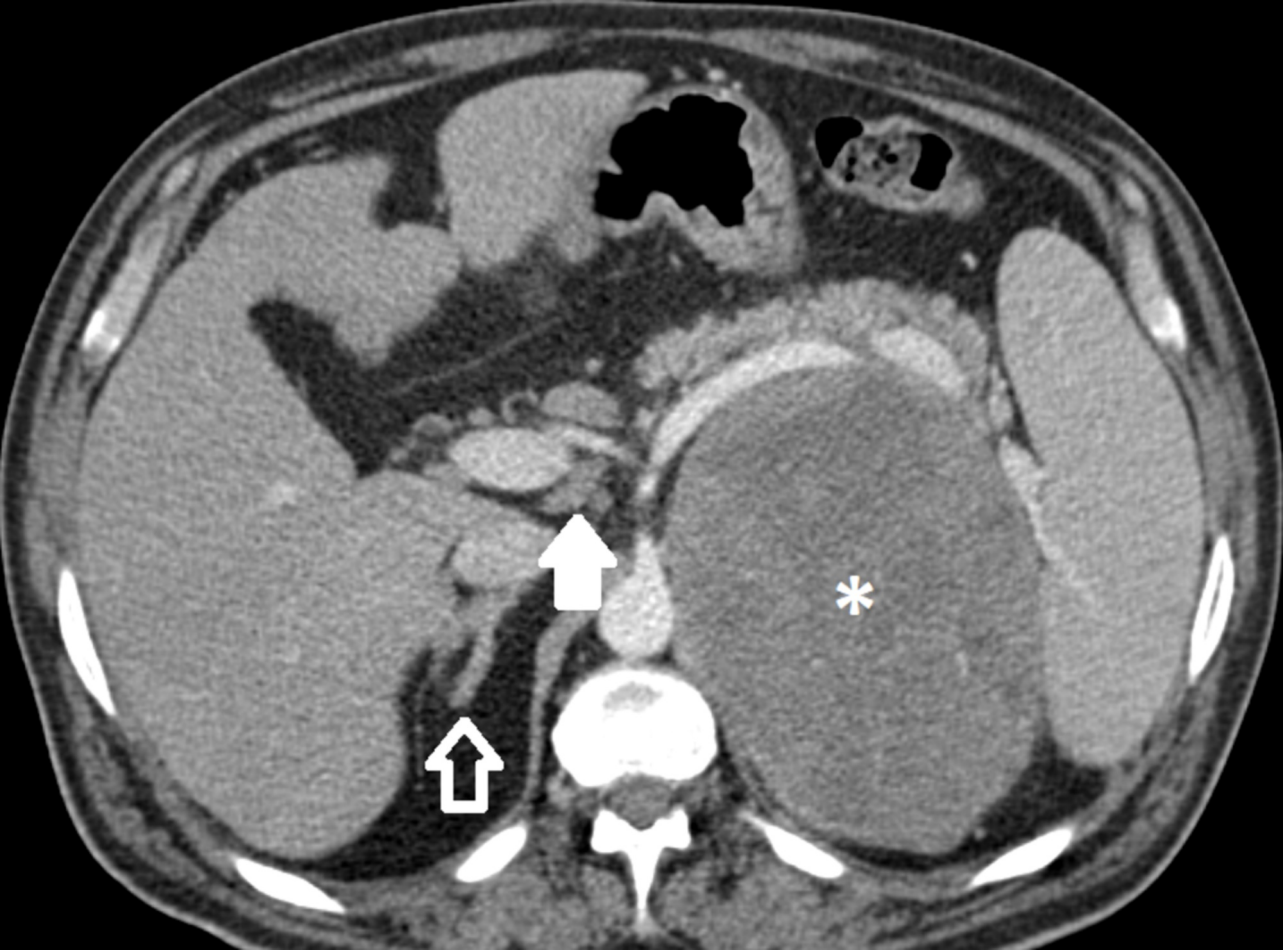
Contrast-enhanced computed tomography (CT) Axial images show a large adrenal mass (asterisk) with a focus of calcification (not shown) arising from the left adrenal gland, measuring higher than 10 Hounsfield units in density. There is local mass effect on the pancreas and spleen. The normal right adrenal gland is pictured for comparison (open arrow). A few sub-centimeter retroperitoneal lymph nodes are present (solid arrow).

A 58-year-old man with a history of type one diabetes mellitus presented to his primary care physician with hematochezia, left-sided abdominal pain, and fatigue for two months. A complete blood count was significant for anemia with a hemoglobin of 8.8 g/dL. He was subsequently admitted to an outside institution where he underwent a colonoscopy which revealed two sigmoid polyps consistent with tubular adenoma and a hyperplastic polyp. One week following discharge, he continued to have left-sided abdominal pain which prompted admission to our institution. Here, a computed tomography (CT) scan of his abdomen revealed a large, heterogeneous, partially necrotic retroperitoneal mass measuring 15 cm x 10.9 cm x 13.9 cm arising from the left adrenal gland as well as prominent retroperitoneal lymph nodes (Figure 1). The mass displaced the left kidney, renal vessels, pancreas, and splenic vein. A CT guided biopsy revealed a poorly-differentiated malignant neoplasm with neuroendocrine features. Work-up with plasma renin, aldosterone, random cortisol, adrenocorticotropic hormone (ACTH), ACTH stimulation test, chromogranin-A, and urinary catecholamines was unrevealing. Urinary cortisol and neuron-specific enolase were only mildly elevated. 

He then underwent an exploratory laparotomy with left adrenalectomy, left nephrectomy, splenectomy, partial gastrectomy, and distal pancreatectomy. The pathology revealed a malignant tumor sized 17.5 cm, weight 1175 g with evidence of microscopic extravascular extension and lymphovascular invasion. The tumor was positive for vimentin, factor 8, CD34, CD31, and synaptophysin and negative for cytokeratin chromogranin, CD68, S100, desmin, and actin. Upon consultation with pathology from two other institutions, it was determined that the tumor was a poorly differentiated malignant neoplasm consistent with epithelioid adrenal angiosarcoma.

Two months following surgery, the patient was started on adjuvant chemotherapy with paclitaxel. A re-staging positron emission tomography (PET) scan following two cycles of therapy showed several hypermetabolic soft tissue nodules in the surgical bed and left retroperitoneum concerning for disease recurrence. Due to the presence of only one viable kidney following his nephrectomy, ifosfamide was avoided and he was subsequently started on palliative chemotherapy with single-agent doxorubicin. A PET scan following three cycles of doxorubicin therapy revealed interval disease progression in his surgical bed and metastatic disease in the abdominal soft tissue inferior to the stomach as well as to the right adrenal gland. His chemotherapy regimen was then switched to gemcitabine with vinorelbine for three cycles. He continued to have disease progression on scans and decided to transfer his care to another institution, where he was later started on carboplatin therapy. However, CT imaging following two cycles of carboplatin therapy showed extensive retroperitoneal metastatic disease progression with a new mass in the distal colon. Because he did not show any response to these four lines of therapy generally used for angiosarcoma, he was started on etoposide-doxorubicin-mitotane therapy which is typically reserved for advanced adrenal cortical carcinoma [4]. During the nine-month period he was receiving chemotherapy, he was hospitalized three times- once for small bowel obstruction, and two other times for pain control. While getting his fifth line of treatment, his functional status gradually declined and he passed away shortly after.

## Discussion

Adrenal angiosarcoma is a highly uncommon and aggressive malignancy which is difficult to diagnose. It was first described by Kareti et al. in 1988 [5]. It is more common in men, and occurs most frequently in individuals in the sixth and seventh decades of life [6]. Patients typically present with abdominal pain and/or flank pain, though symptoms can also include weight loss, fatigue or weakness or those who are asymptomatic with incidental findings on imaging. Predisposing risk factors for angiosarcomas include a history of familial angiodysplasia, chronic lymphedema, anabolic steroid use and exposure to thorium dioxide or radiation [2]. While the etiology of the majority of case reports of adrenal angiosarcoma is unknown, there have been a few case reports of adrenal angiosarcoma associated with abdominal fibromatosis and exposure to arsenic-containing insecticides and vinyl chloride [3]. CT or magnetic resonance imaging and biopsy with appropriate immunohistochemical staining is used to arrive at the diagnosis.

**Figure 2:**
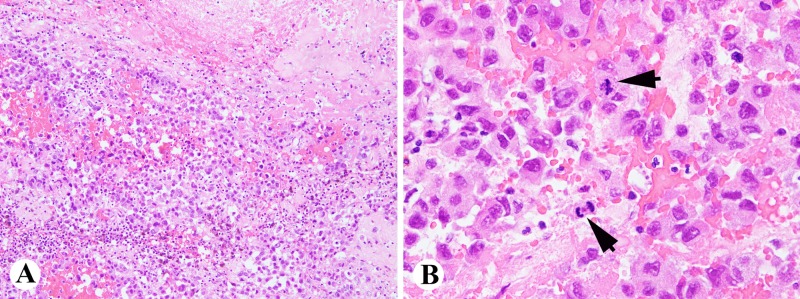
Histologically, the tumor is composed of atypical epithelial cells with hemorrhages, necrosis, and brisk mitotic activity (arrows) Hematoxylin and eosin (H&E) stain A, 100x; B, 400x.

**Figure 3:**
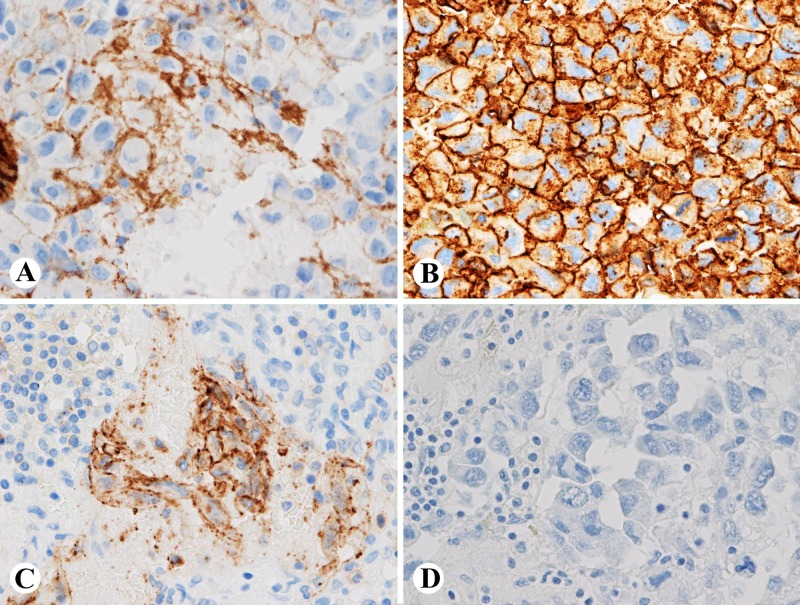
Immunostains of the tumor show that the tumor cells are positive for factor 8 (A), CD34 (B) and focally positive for CD31 (C), negative for AE1/AE3 (D) (A-D, 400x)

However, making the diagnosis of primary adrenal angiosarcoma is challenging for several reasons. First, it is a very rare malignancy and is often not considered in the initial differential diagnosis of an adrenal mass. Therefore, it is important to consider and potentially rule out these other causes which include adrenal cortical carcinoma, pheochromocytoma, adrenal adenomas, primary hyperaldosteronism, lymphoma, metastatic adenocarcinoma, metastatic melanoma or metastatic angiosarcoma. There is often confusion with carcinoma of the adrenal gland, especially when the stains are not conclusive as in this case. It is an important distinction to make, because these diseases are treated differently. Furthermore, it is sometimes difficult to identify on biopsy due to the level of necrosis and hemorrhage found in specimens (Figures 2A-2B) [7]. The cystic changes often observed can be seen with other neoplasias such as adrenal adenomas and pheochromocytomas [8]. In our case, pathology was inconclusive and required outside review to help establish a diagnosis. Also, adrenal angiosarcomas have more of an epithelioid appearance on histology, as opposed to most angiosarcomas that have vasoformative patterns [1]. Finally, an incorrect diagnosis can be made if a wide immunohistochemical panel is not performed. These tumors typically stain positive for cytokeratin, an epithelial tumor marker, which can be seen in metastatic epithelial tumors or other mesenchymal neoplasms [3]. Positive endothelial markers such as CD31, CD34, Factor 8, and FLI1 performed on a wider immunohistochemical panel are needed to confirm the diagnosis of primary adrenal angiosarcoma (Figures 3A-3D) [3,9].

There is no standardized treatment protocol due to the low incidence of this malignancy. Patients with resectable masses usually undergo adrenalectomy. Due to the aggressive and infiltrative capacity of the disease, other visceral organs in the vicinity often need to be removed as was the case of our patient. Median overall survival is higher in those with localized disease following surgery [6]. Adjuvant chemotherapy with adriamycin or paclitaxel with radiation therapy are considerations for individuals with advanced or unresectable disease [10]. Angiosarcomas as a whole have a five-year survival rate of 24 to 31% [10-11]. Though data have shown improved two-year survival in patients with visceral angiosarcoma treated by surgery and adjuvant chemotherapy, the overall prognosis is often poor [6]. Our patient had extensive metastatic disease, and did not respond to multiple lines of palliative chemotherapy following his surgery. Treatment of this malignancy, like its diagnosis, remains challenging. A multidisciplinary approach through the expertise of medical oncology, surgical oncology, and pathology is needed for the management of this disease. Unfortunately, at the time of discovery of his tumor, our patient had several poor prognostic factors including tumor size greater than 5 centimeters, visceral location, tumor necrosis, and likely metastatic disease at presentation, which did not portend a favorable outcome [12].

## Conclusions

Our case highlights the importance of using appropriate laboratory tests and immunohistochemical studies to differentiate between the various types of adrenal masses. Treatment of this malignancy, like its diagnosis, remains challenging due to the absence of a standardized treatment protocol and the often aggressive capability of this disease. A multidisciplinary approach through the expertise of medical oncology, surgical oncology, and pathology is essential for disease management.
